# The nuclear hormone receptor NHR-86 controls anti-pathogen responses in *C*. *elegans*

**DOI:** 10.1371/journal.pgen.1007935

**Published:** 2019-01-22

**Authors:** Nicholas D. Peterson, Hilary K. Cheesman, Pengpeng Liu, Sarah M. Anderson, Kyle J. Foster, Richa Chhaya, Paola Perrat, Jose Thekkiniath, Qiyuan Yang, Cole M. Haynes, Read Pukkila-Worley

**Affiliations:** 1 Program in Innate Immunity, Division of Infectious Diseases and Immunology, University of Massachusetts Medical School, Worcester, MA, United States of America; 2 Department of Molecular, Cell and Cancer Biology, University of Massachusetts Medical School, Worcester, MA, United States of America; 3 Department of Neurobiology, University of Massachusetts Medical School, Worcester, MA, United States of America; The University of Texas Health Science Center at Houston, UNITED STATES

## Abstract

Nuclear hormone receptors (NHRs) are ligand-gated transcription factors that control adaptive host responses following recognition of specific endogenous or exogenous ligands. Although NHRs have expanded dramatically in *C*. *elegans* compared to other metazoans, the biological function of only a few of these genes has been characterized in detail. Here, we demonstrate that an NHR can activate an anti-pathogen transcriptional program. Using genetic epistasis experiments, transcriptome profiling analyses and chromatin immunoprecipitation-sequencing, we show that, in the presence of an immunostimulatory small molecule, NHR-86 binds to the promoters of immune effectors to activate their transcription. NHR-86 is not required for resistance to the bacterial pathogen *Pseudomonas aeruginosa* at baseline, but activation of NHR-86 by this compound drives a transcriptional program that provides protection against this pathogen. Interestingly, NHR-86 targets immune effectors whose basal regulation requires the canonical p38 MAPK PMK-1 immune pathway. However, NHR-86 functions independently of PMK-1 and modulates the transcription of these infection response genes directly. These findings characterize a new transcriptional regulator in *C*. *elegans* that can induce a protective host response towards a bacterial pathogen.

## Author summary

The family of nuclear hormone receptors (NHRs) has expanded in the nematode *C*. *elegans*. NHRs are intracellular sensors that control adaptive host responses following recognition of specific endogenous or exogenous ligands. These proteins are therefore well positioned to function in pathogen sensing and innate immune activation. The *C*. *elegans* genome contains 284 NHRs, whereas humans and *Drosophila* have only 48 and 21, respectively. However, the biological function of the great majority of the NHRs in *C*. *elegans* is not known. We designed a genetic screen to determine if an NHR functions in immune activation, and identified NHR-86, a homolog of human hepatocyte nuclear factor 4 (HNF4). We show that NHR-86 drives a transcriptional response that provides protection against the bacterial pathogen *Pseudomonas aeruginosa*.

## Introduction

Nuclear hormone receptors (NHRs) are transcription factors that regulate a number of key biological processes following recognition of specific exogenous or endogenous ligands. Interestingly, the genomes of *Caenorhabditis* species contain a large number of NHRs compared to other metazoans [1]. 284 NHRs are present in *C*. *elegans*, whereas *Drosophila* and humans have only 21 and 48, respectively [2]. The marked expansion of NHRs suggests that these proteins play particularly important roles in nematode physiology [2, 3]; however, only a very small minority of *C*. *elegans* NHRs have been characterized in detail [2].

Like other metazoans, *C*. *elegans* rely on inducible host defense mechanisms during infection with bacterial pathogens [4–7]. The mechanisms that engage these immune defenses are not completely understood. Considering their roles as intracellular sensors of specific ligands, we hypothesized that NHRs function in innate immune activation in *C*. *elegans*. However, forward genetic screens did not previously identify an NHR that is necessary for pathogen resistance [8, 9]. We, therefore, designed a genetic screen to determine if an NHR could activate protective immune defenses in *C*. *elegans*.

Utilizing a potent immunostimulatory small molecule as a chemical probe, we identified NHR-86 and showed that it drives a transcriptional response that protects *C*. *elegans* from infection with the bacterial pathogen *Pseudomonas aeruginosa*. NHR-86 is a homolog of mammalian hepatocyte nuclear factor 4 (HNF4), an NHR that has been implicated in the pathogenesis of inflammatory bowel disease [10–13]. We show that, in the presence of an immunostimulatory small molecule, NHR-86 induces innate immune defenses by binding to the promoters of immune effectors, in a manner that does not require the canonical p38 MAPK PMK-1 pathway. In this context, PMK-1 sets the basal expression of innate immune response genes, but is dispensable for their induction by NHR-86. These data demonstrate a new mechanism by which immune defenses are engaged to protect the worm and raise the possibility that the expansion of the NHR family in *C*. *elegans* may have been fueled, at least in part, by the roles of these proteins in the activation of host defense responses.

## Results

### An RNAi screen identifies a role for the nuclear hormone receptor *nhr-86* in the induction of *C*. *elegans* immune effectors

**Fig 1:**
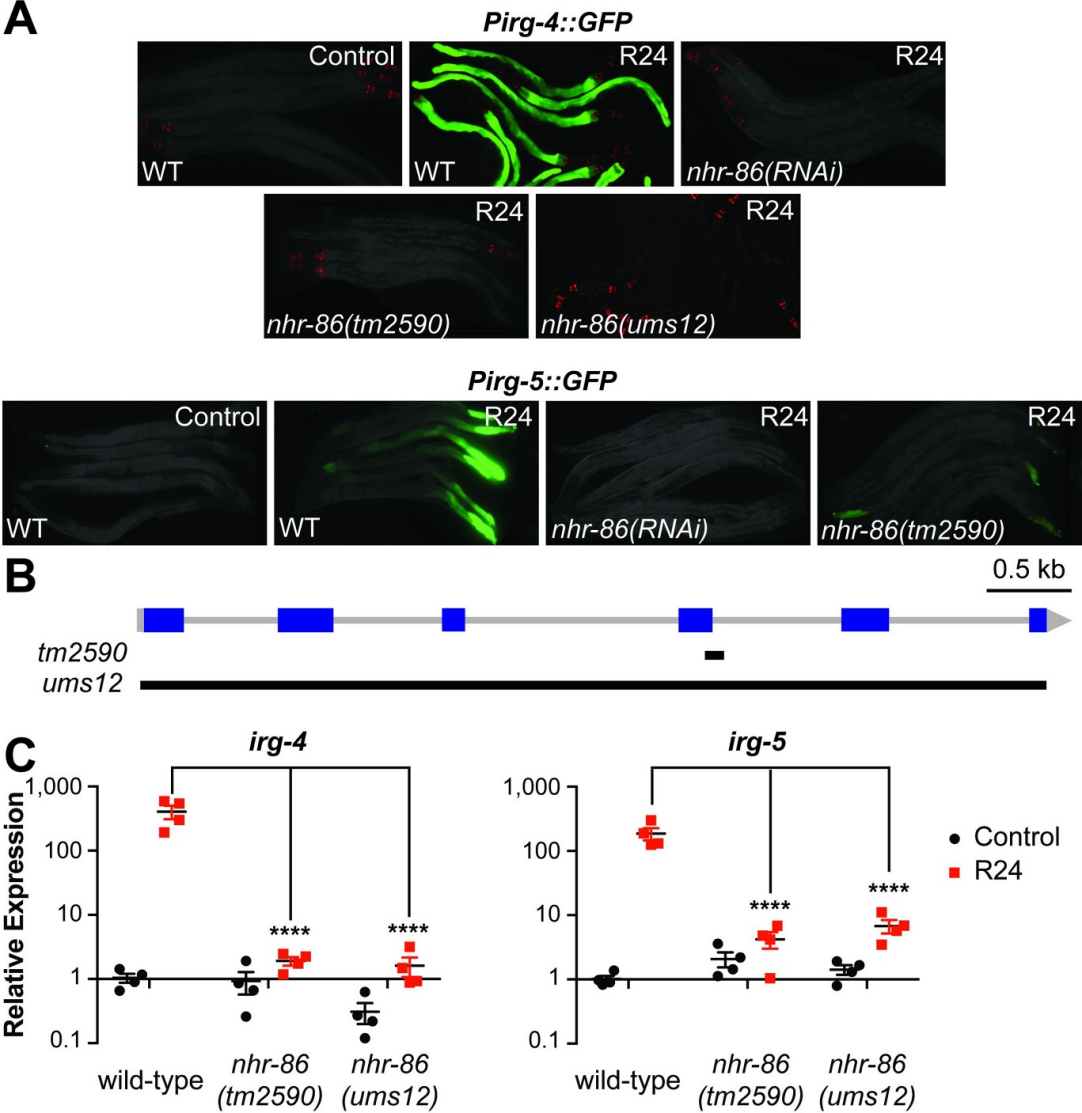
An RNAi screen identifies a role for the nuclear hormone receptor *nhr-86* in the induction of *C*. *elegans* immune effectors. **(A)** *C*. *elegans* carrying either the *Pirg-4*(F08G5.6)::*GFP* or the *Pirg-5*(F35E12.5)::*GFP* immune reporter of the indicated genotypes were transferred at the L4 stage to media supplemented with either R24 or the solvent control (1% DMSO) for approximately 18 hours. Red pharyngeal expression is the *Pmyo-2*::*mCherry* co-injection marker, which confirms the presence of the *Pirg-4*::*GFP* transgene. Presence of the *Pirg-5*::*GFP* transgene was confirmed by assaying for the Rol phenotype. Photographs were acquired using the same imaging conditions for each immune reporter. **(B)** Model of the *nhr-86* gene. Blue squares are exons. Black lines show the locations of the deletions in each of the *nhr-86* mutants. **(C)** The expression of the *C*. *elegans* immune effector genes *irg-4*, *irg-5* and *irg-6* (C32H11.1) were analyzed by qRT-PCR in wild-type animals and in two different *nhr-86* loss-of-function mutants (*tm2590* and *ums12*), each exposed to either R24 or control for approximately 18 hours. Data for *irg-6* is shown in S1B Fig. Data are the average of four independent replicates, each normalized to a control gene with error bars representing SEM. Data are presented as the value relative to the average expression from all replicates of the indicated gene in the baseline condition (wild-type animals exposed to control). The difference in induction of *irg-4*, *irg-5* and *irg-6* by R24 in wild-type animals compared to each of the two *nhr-86* mutant strains is significant (**** p<0.0001 by 2-way ANOVA with Bonferroni multiple comparisons test).

To determine if an NHR can induce protective immune responses, 258 of the 284 NHR genes in the *C*. *elegans* genome were screened by RNAi [14] using the *C*. *elegans Pirg-4*(F08G5.6)::*GFP* transcriptional immune reporter and the immunostimulatory xenobiotic R24. R24 (also referred to as RPW-24) was originally identified in a screen of 37,214 small molecules for new anti-infective compounds [15]. This molecule robustly activates innate immune defenses and protects nematodes infected with bacterial pathogens [7, 16–18]. For this screen, the *Pirg-4*::*GFP* transcriptional reporter was chosen as a convenient readout of immune activation. IRG-4 (infection response gene-4) contains a CUB-like domain, a group of secreted proteins that are postulated to play a role in host defense [19]. Basal levels of *irg-4* transcription are controlled by the p38 MAPK PMK-1 pathway [19]. This gene is induced during infection by multiple bacterial pathogens, including *P*. *aeruginosa* [19–23], and by the small molecule R24 [16–18]. RNAi-mediated knockdown of ten NHRs partially affected the R24-mediated induction of *Pirg-4*::*GFP* by R24 (S1 Table), but only one NHR (*nhr-86*) completely abrogated the upregulation of this immune reporter (Fig 1A).

We confirmed the results of the *nhr-86(RNAi)* experiment using several approaches. The previously characterized null allele *nhr-86(tm2590)* [3], which contains a 172 bp deletion that removes 33 bp in exon 4 of *nhr-86*, suppressed *Pirg-4*::*GFP* induction by R24 (Fig 1A and 1C). CRISPR-Cas9 was used to generate a clean deletion of *nhr-86* [*nhr-86(ums12)*] (Fig 1B). *ums12* is a 5.5 kb deletion that removes nearly all of the *nhr-86* coding region, which caused a marked reduction in the *nhr-86* transcript (S1A Fig). The *nhr-86(ums12)* mutation fully suppressed the induction of *Pirg-4*::*GFP* by R24 (Fig 1A and 1C).

In addition to *irg-4*, *nhr-86* is required for the R24-dependent transcriptional upregulation of two additional immune effectors that contain CUB-like domains, *irg-5* (F35E12.5) and *irg-6* (C32H11.1) (Figs 1 and S1B). Like *irg-4*, *irg-5* and *irg-6* are induced by several different bacterial pathogens, require the p38 MAPK PMK-1 pathway for their basal transcriptional levels, and are induced in accordance with the virulence potential of the pathogen [17, 19–24]. R24-mediated induction of the *Pirg-5*::*GFP* transgene was abrogated by *nhr-86(RNAi)* and in the *nhr-86(tm2590)* background (Fig 1A). In addition, qRT-PCR of *irg-5* and *irg-6* showed that *nhr-86* loss-of-function mutations suppress induction by R24 (Figs 1C and S1B).

Interestingly, RNAi-mediated knockdown of *irg-4* renders worms hypersusceptible to killing by *P*. *aeruginosa* [25, 26]. Importantly, *irg-4* knockdown does not shorten the lifespan of nematodes growing on *E*. *coli* OP50, the normal laboratory food source, nor does its knockdown cause susceptibility to other stressors [25, 26]. We confirmed these observations and also found that *irg-5(RNAi)* and *irg-6(RNAi)* animals are more susceptible to killing by *P*. *aeruginosa* (S1C Fig). As with *irg-4(RNAi)*, knockdown of *irg-5* or *irg-6* did not shorten the lifespan of *C*. *elegans* growing on *E*. *coli* OP50 (S1D Fig). Thus, *nhr-86* drives the induction of at least three innate immune effectors that confer resistance to *P*. *aeruginosa* infection.

### *nhr-86* activates the transcription of innate immune response genes

**Fig 2:**
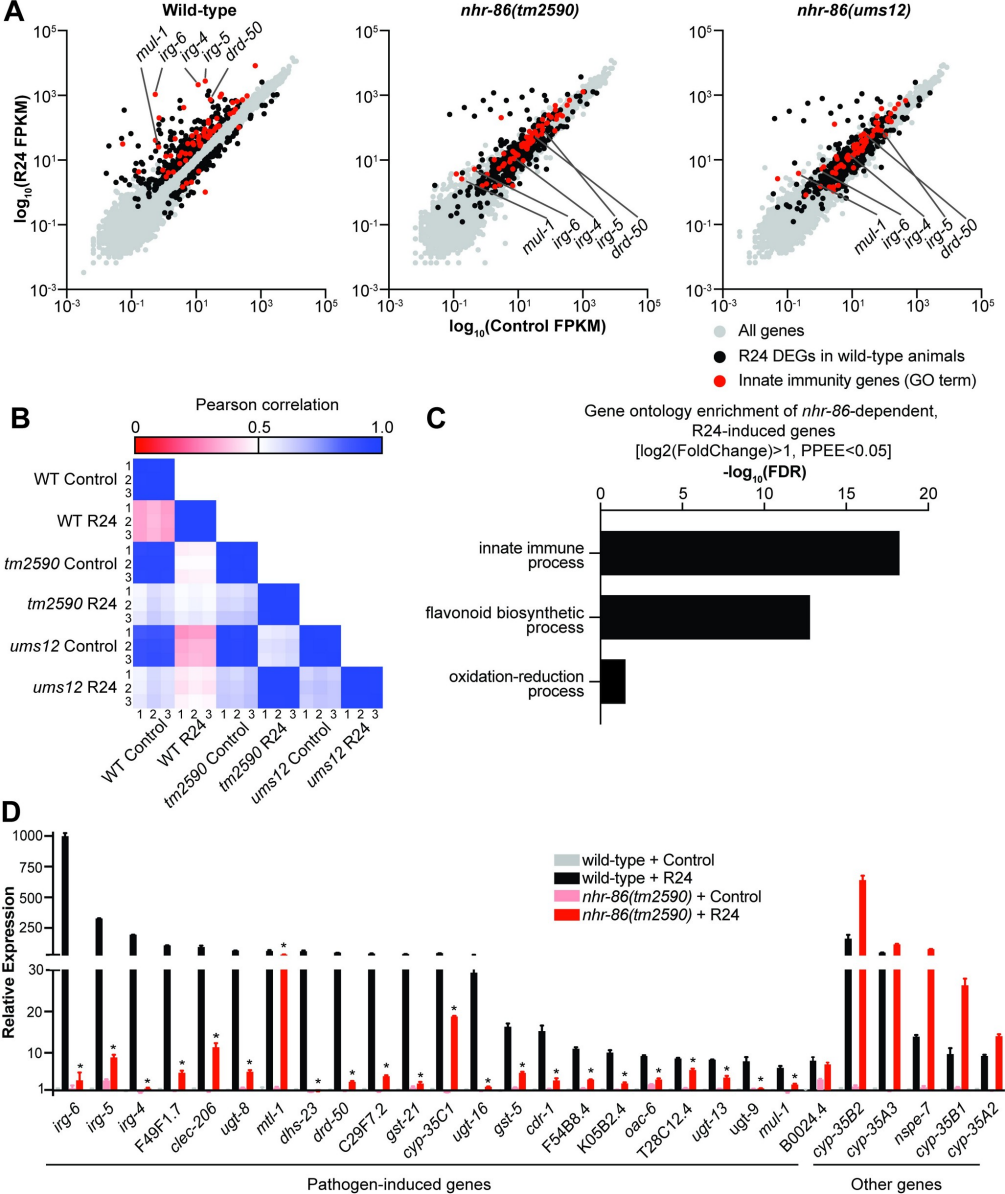
*nhr-86* activates the transcription of innate immune response genes. **(A)** All data from the mRNA-seq experiment are presented on scatter plots. Genes that were differentially regulated upon R24 treatment in wild-type animals are shown in black (Fold change> 2, PPEE<0.05). These same genes are also highlighted in black in the *nhr-86(tm2590)* and *nhr-86(ums12)* scatter plots. Genes involved in innate immunity by Gene Ontology (GO) term are highlighted in red. **(B)** Pearson correlation coefficients are presented for all samples in the mRNA-seq experiment. **(C)** Gene ontology enrichment of the *nhr-86*-dependent, R24-induced genes identified in the mRNA-seq experiment are shown. **(D)** Results of NanoString nCounter gene expression analysis for 118 *C*. *elegans* genes performed on wild-type and *nhr-86(tm2590)* animals exposed to either R24 or control. The 28 genes that were induced 5-fold or greater in wild-type animals by R24 are presented. Data are the average of three replicates, each of which was normalized to three control genes, with error bars representing standard deviation and are presented as the value relative to the average expression from the replicates of the indicated gene in the baseline condition (wild-type animals exposed to control). * p<0.05 by student’s t*-*test for the comparison of the R24-induced conditions.

To define the genes that are dependent on *nhr-*86 for their transcription, we performed mRNA-sequencing. We compared the mRNA expression profiles of wild-type animals and two different *nhr-86* loss-of-function alleles (*tm2590* and *ums12*), each exposed to the immunostimulatory molecule R24 or mock treatment. Exposure to R24 caused the induction of 391 genes, which (as in previous studies) were enriched for innate immune response and xenobiotic detoxification genes [16–18]. The upregulation of 147 of these genes in the *nhr-86*(*tm2590)* mutants and 205 genes in the *nhr-86(ums12)* mutants were significantly attenuated (Fig 2A). Importantly, the mRNA expression patterns of both *nhr-86* loss-of-function mutants were tightly correlated (Fig 2B) with 142 misregulated genes in common between these two datasets (S2 Table). Analysis of these 142 *nhr-86*-dependent genes revealed a significant enrichment of innate immune genes and those involved in the defense response to bacterial pathogens (Fig 2A and 2C). Included among these *nhr-86-*dependent genes are the representative immune effectors *irg-4*, *irg-5*, *irg-6*, *mul-1*(F49F1.6) and *drd-50*(F49F1.1) (Fig 2A). *mul-1* and *drd-50* are induced during infection with multiple bacterial pathogens, including *P*. *aeruginosa* [17, 19–24].

To confirm the results of our mRNA-seq data, we used a NanoString codeset to examine the expression of 118 innate immune and stress response genes in biological replicate RNA samples from wild-type and *nhr-86(tm2590)* animals (Fig 2D). From the NanoString codeset, we identified 28 genes induced by R24, 23 of which were pathogen-response genes. Of the 23 pathogen-response genes, we identified 22 that are dependent on *nhr-86* for their induction. The NanoString experiment also confirmed the observation in the mRNA-seq experiment that *nhr-86* is not required for the induction of all R24-induced genes (Fig 2A and 2D). Interestingly, many of these genes that are upregulated by R24 in a manner independent of *nhr-86* are cytochrome P450s, which are involved in the detoxification of xenobiotics (Fig 2D and S2 Table). Thus, *nhr-86* is required for the upregulation of only a specific subset of the R24-induced genes, a group that is strongly enriched for innate immune effectors (Fig 2C).

Interestingly, examination of the mRNA-seq profiles of *C*. *elegans* that were not exposed to compound (*i*.*e*., normal growth conditions or basal expression) revealed that the expression of 302 genes were significantly lower in the *nhr-86* loss-of-function mutants compared to wild-type animals (>2-fold change, PPEE<0.05) and only 11 of these genes were differentially regulated more than 4-fold (PPEE<0.05). Only 6 of these genes were among the 142 genes that required *nhr-86* for their induction by R24. Comparison of the basal expression of *irg-4*, *irg-5* and *irg-6* in the two *nhr-86* loss-of-function alleles with wild-type animals by qRT-PCR confirmed this observation (Fig 1C and S1B Fig). Thus, while *nhr-86* is necessary for the transcriptional induction of genes and innate immune effectors in particular, it is largely dispensable for their basal regulation.

### NHR-86 binds to the promoters of innate immune genes to drive their transcription

**Fig 3:**
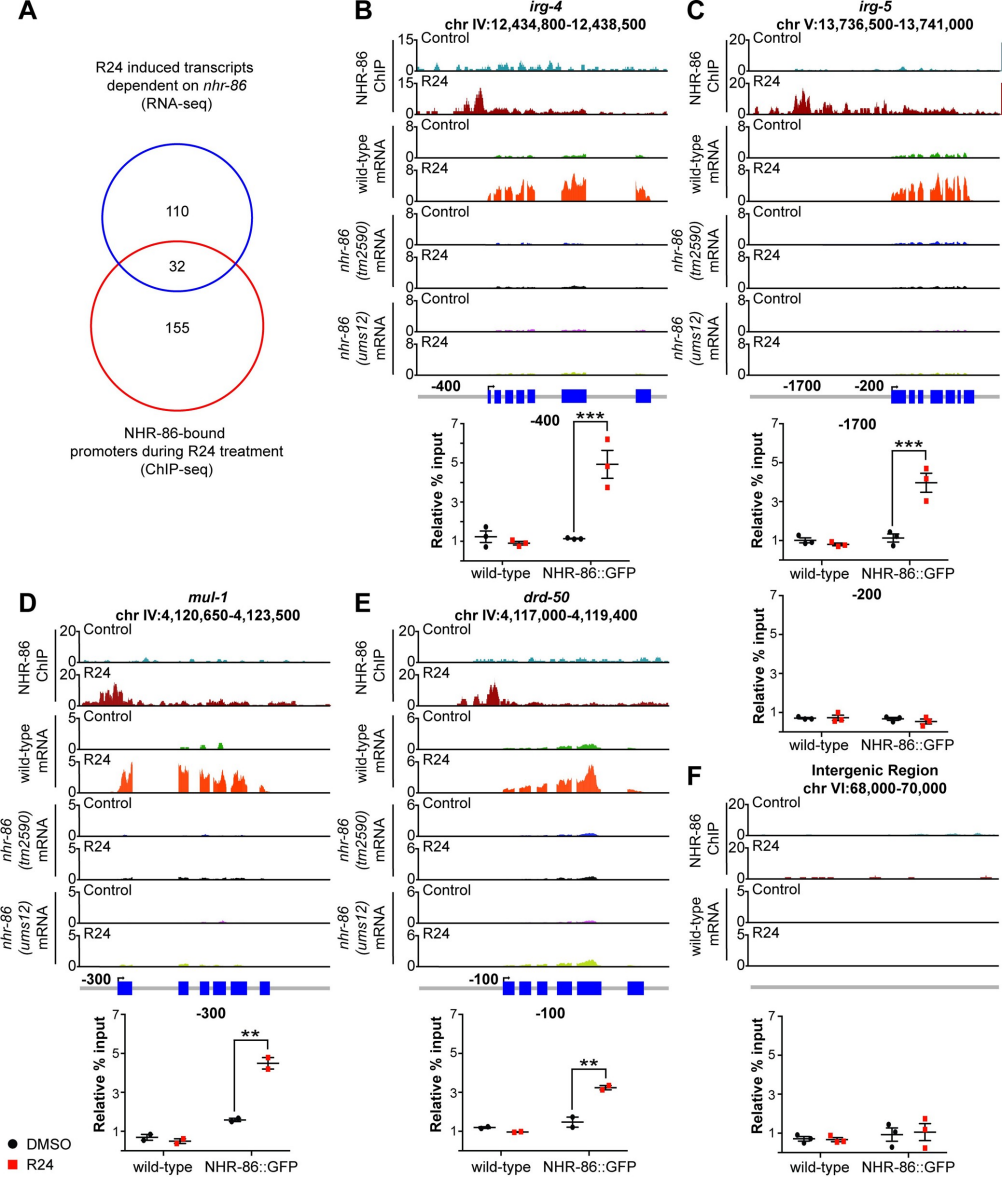
NHR-86 binds to the promoters of innate immune genes to drive their transcription. **(A)** Venn diagram showing the number of *nhr-86-*dependent, R24-induced genes in the mRNA-seq experiment, the genes whose promoters were bound by NHR-86 following R24 treatment in the ChIP-seq experiment, and the overlap between these datasets. The overlap between these datasets is significantly more than is expected by chance alone (1.1 gene overlap expected by chance, hypergeometric p-value = 2x10^-39^). ChIP-seq profiles, mRNA-seq profiles and confirmatory ChIP-PCR are presented for the representative immune effectors *irg-4* **(B),** *irg-5* **(C),** *mul-1* (F49F1.6) **(D)** and *drd-50* (F49F1.1) **(E)** in animals of the indicated genotype exposed to R24 or the control. The y-axis is the number of reads (log_2_). A gene model shows the location of the exons (blue) of the indicated genes. ChIP was performed with an anti-GFP antibody in *C*. *elegans* wild-type and transgenic NHR-86::GFP animals. Final set of peaks were called if the difference in intensity values of samples had a significance level of p-value < 0.025 (see S3 Table) for the indicated comparison. In the ChIP-PCR data, the percent input for each condition was normalized to the abundance of a random intergenic region of chromosome four. ** p<0.01 and *** p<0.001 by 2-way ANOVA with Bonferroni multiple comparisons test for the indicated comparison. A region 200 bp upstream of *irg-5* **(C)** and a random intergenic region on chromosome six **(F)** were not enriched by control or R24 treatment. Each data point in the ChIP-qPCR data is from an independent biological replicate.

To determine the direct targets of NHR-86 during R24 exposure, we performed chromatin immunoprecipitation-sequencing (ChIP-seq). Of the 142 genes that are induced by R24 in an *nhr-86*-dependent manner, NHR-86 bound to the promoters of 32 of these genes following treatment with R24 compared to control (Fig 3A and S3 Table). All but one of these 32 genes are induced during infection with at least one bacterial pathogen, including 14 genes that are upregulated during infection with *P*. *aeruginosa* (S3 Table). Among the immune effectors whose transcription is directly regulated by NHR-86 are *irg-4* (Fig 3B), *irg-5* (Fig 3C), *mul-1* (Fig 3D), *drd-50* (Fig 3E) and *irg-6* (S3 Table). The ChIP-seq experiment was performed with a strain containing a GFP-tagged NHR-86 protein (NHR-86::GFP) that has been previously characterized [3]. The induction of *irg-4* by R24 was restored in *nhr-86(tm2590)* mutants, which contained this NHR-86::GFP construct (S2A Fig).

ChIP followed by qPCR (ChIP-qPCR) was used to confirm that NHR-86 binds to the promoters of innate immune effectors following R24 treatment. Promoter regions associated with *irg-4* (Fig 3B), *irg-5* (Fig 3C), *mul-1* (Fig 3D) and *drd-50* (Fig 3E) were significantly enriched following R24 treatment, but not in samples exposed to the solvent control. In addition, these promoter regions were not enriched in either control or R24-exposed wild-type animals, which do not express NHR-86::GFP that was used to immunoprecipitate promoter fragments. Binding of NHR-86 to the promoters of immune response genes upon R24 treatment was associated with a corresponding increase in mRNA transcript levels of these genes, which was entirely abrogated in both *nhr-86* loss-of-function mutants (Fig 3B–3E). Importantly, a control region within the *irg-5* promoter (Fig 3C) and a random intergenic region on chromosome VI (Fig 3F) were not enriched in the ChIP-qPCR or ChIP-seq experiments. In addition, 110 genes were induced by R24 in an *nhr-86*-dependent manner, but NHR-86 did not bind to their promoters. Of note, NHR-86 is expressed in the nuclei of *C*. *elegans* intestinal epithelial cells [3] and promotes the induction of the innate immune effectors *irg-4*::*GFP* and *irg-5*::*GFP* in the intestine (Fig 1A), the tissue that directly interfaces with ingested pathogens.

A motif analysis was performed on the promoters bound by NHR-86::GFP to identify putative regulatory sequences. A single 15 bp sequence was strongly enriched in these promoters (E-value: 1.7e-003, S2C Fig). 15 of the 32 genes whose transcription were directly regulated by NHR-86 in the presence of R24 contain this 15 bp element in their promoters, including *irg-4*, *irg-5* and *mul-1* (S3 Table). However, only 3 of 172 genes that are induced by R24 independent of NHR-86 contain this 15 bp element. These data suggest that this 15 bp sequence may be a potential binding site for NHR-86.

Together, the mRNA-seq and ChIP-seq data demonstrate that, in the presence of an immunostimulatory molecule, NHR-86 engages the promoters of innate immune effector genes to drive their transcription. Under normal growth conditions, *nhr-86* does not bind to the promoters of immune effectors (*e*.*g*., *irg-4*, *irg-5*, *mul-1* and *drd-50*) and does not affect their basal expression. These data are the first demonstration of direct immune effector regulation by a nuclear hormone receptor in *C*. *elegans*.

### The immune response induced by *nhr-86* protects *C*. *elegans* from *P*. *aeruginosa* infection

**Fig 4:**
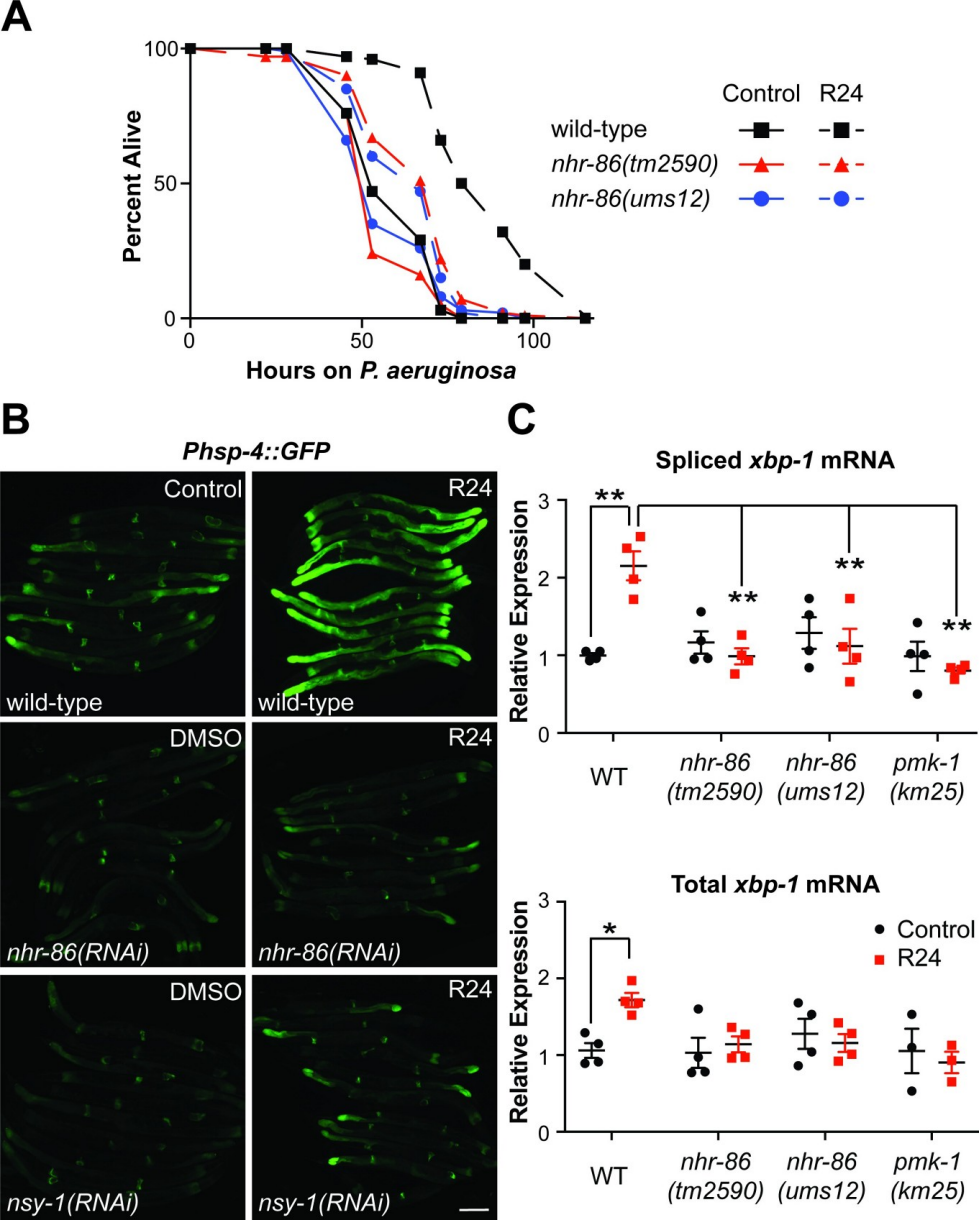
The immune response induced by *nhr-86* protects *C*. *elegans* from *P*. *aeruginosa* infection. **(A)** *P*. *aeruginosa* infection assays of *C*. *elegans* wild-type, *nhr-86(tm2590)* and *nhr-86(ums12)* treated with R24 or 1% DMSO (control) are shown. The difference in susceptibility to *P*. *aeruginosa* between R24-exposed wild-type and each of the mutant animals is significant (p<0.001 by the log-rank test). Data are representative of three trials. Sample sizes, mean lifespan, % lifespan extension conferred by R24 treatment in each background and p values for all trials are shown in S4A Table. Significance was determined using Kaplan-Meier survival curves and log-rank tests. **(B)** *C*. *elegans* carrying the *Phsp-4*::*GFP* reporter were exposed to the indicated RNAi bacteria and transferred at the L4 stage to media supplemented with either R24 or control for approximately 18 hours. Scale bar equals 100 μm. **(C)** qRT-PCR was used to measure the spliced (active) and total *xbp-1* mRNA in animals of the indicated genotype exposed to R24 or control. Comparisons were calculated using 2-way ANOVA with Bonferroni multiple comparisons test and * p<0.05 and ** p<0.0001. Data are the average of four independent replicates, each normalized to a control gene with error bars representing SEM. Data are presented as the value relative to the average expression from all replicates of the indicated gene in the baseline condition (wild-type animals exposed to control).

To determine if *nhr-86* induces a physiologically-relevant transcriptional response, we compared the susceptibility of the *nhr-86* loss-of-function mutants to *P*. *aeruginosa* infection following exposure to R24. R24 protects wild-type *C*. *elegans* during *P*. *aeruginosa* infection [16–18]. Consistent with the key role of *nhr-86* in driving the induction of innate immune defenses, *nhr-86* loss-of-function mutants (*tm2590* and *ums12*) significantly suppressed the pathogen-resistance phenotype of R24-exposed wild-type worms (Fig 4A). Together, these data demonstrate that the defense response induced by *nhr-86* promotes host resistance to bacterial infection.

An alternate method of examining the physiological relevance of immune effector induction in *C*. *elegans* involves studying the effect of induced transcriptional responses on stress in the endoplasmic reticulum (ER). The induction of host immune effectors in *C*. *elegans* requires compensatory activation of the unfolded protein response (UPR) in the ER, presumably to handle the increase in proteins trafficking through this organelle [27, 28]. Accordingly, R24 exposure caused the induction of *Phsp-4*::*GFP*, a transcriptional reporter for the BiP/GRP78 homolog in *C*. *elegans*, which indicates UPR activation (Fig 4B). *hsp-4* transcription is regulated by the transcription factor XBP-1, which is activated by the ER-transmembrane protein IRE-1 when unfolded proteins accumulate in the ER. IRE-1 has RNase activity, which upon activation, cleaves *xbp-1* mRNA to change its reading frame and encode the active XBP-1 protein [29]. We found that exposure to R24 increased the active, spliced form of *xbp-1* (Fig 4C). Total *xbp-1* mRNA was also increased following R24 treatment (Fig 4C). Interestingly, knockdown of *nhr-86* suppressed *Phsp-4*::*GFP* induction (Fig 4B) and the accumulation of active *xbp-1* (Fig 4C) following exposure to the xenobiotic R24. In addition, animals deficient in *nsy-1*, the MAPKKK upstream of the p38 MAPK *pmk-1* (Fig 4B), and *pmk-1* (S3A Fig), failed to induce the *Phsp-4*::*GFP* following exposure to R24. *pmk-1(km25)* mutants abrogated the cleaving of *xbp-1* into its active form (Fig 4C). Thus, R24-mediated immune induction activates the UPR, in a manner dependent on *nhr-86* and the p38 MAP *pmk-1* pathway.

We considered the possibility that R24 is a direct poison of the ER. However, tunicamycin, a potent inducer of ER stress and the UPR, did not activate the immune reporter *Pirg-4*::*GFP* (S3B Fig). In addition, RNAi-mediated knockdown of *nhr-86* did not suppress *Phsp-4*::*GFP* induction by tunicamycin (S3C Fig). Thus, ER stress itself does not lead to the induction of *nhr-86*-dependent innate immune responses, but rather occurs as a consequence of mobilizing this protective host response. These data are consistent with prior reports, which demonstrate that activation of the p38 MAPK *pmk-1* pathway is not dependent on IRE-1/XBP-1 [27, 28]. Together, these data demonstrate that the immune response induced by *nhr-86* following exposure to R24 is a physiologically-relevant source of ER stress and provide further support for the conclusion that *nhr-86* activates a pathogen-defense response involving secreted proteins.

In the absence of R24, *C*. *elegans nhr-86* mutants are not more susceptible to *P*. *aeruginosa* infection than wild-type animals (Fig 4A). In addition, the induction of the innate immune effectors *irg-5*, *irg-6* and *irg-1* during *P*. *aeruginosa* infection is not attenuated in the *nhr-86(ums12)* mutant; however, the induction of *irg-4* is significantly lower (S4 Fig). Given the marked expansion of the NHR family in *C*. *elegans*, NHRs, or potentially another mechanism, may function redundantly with NHR-86 to activate host defense genes during *P*. *aeruginosa* infection. It is also possible that *P*. *aeruginosa* does not produce the ligand sensed by NHR-86.

### *nhr-86* induces innate immune defenses independent of the p38 MAPK *pmk-1*

**Fig 5:**
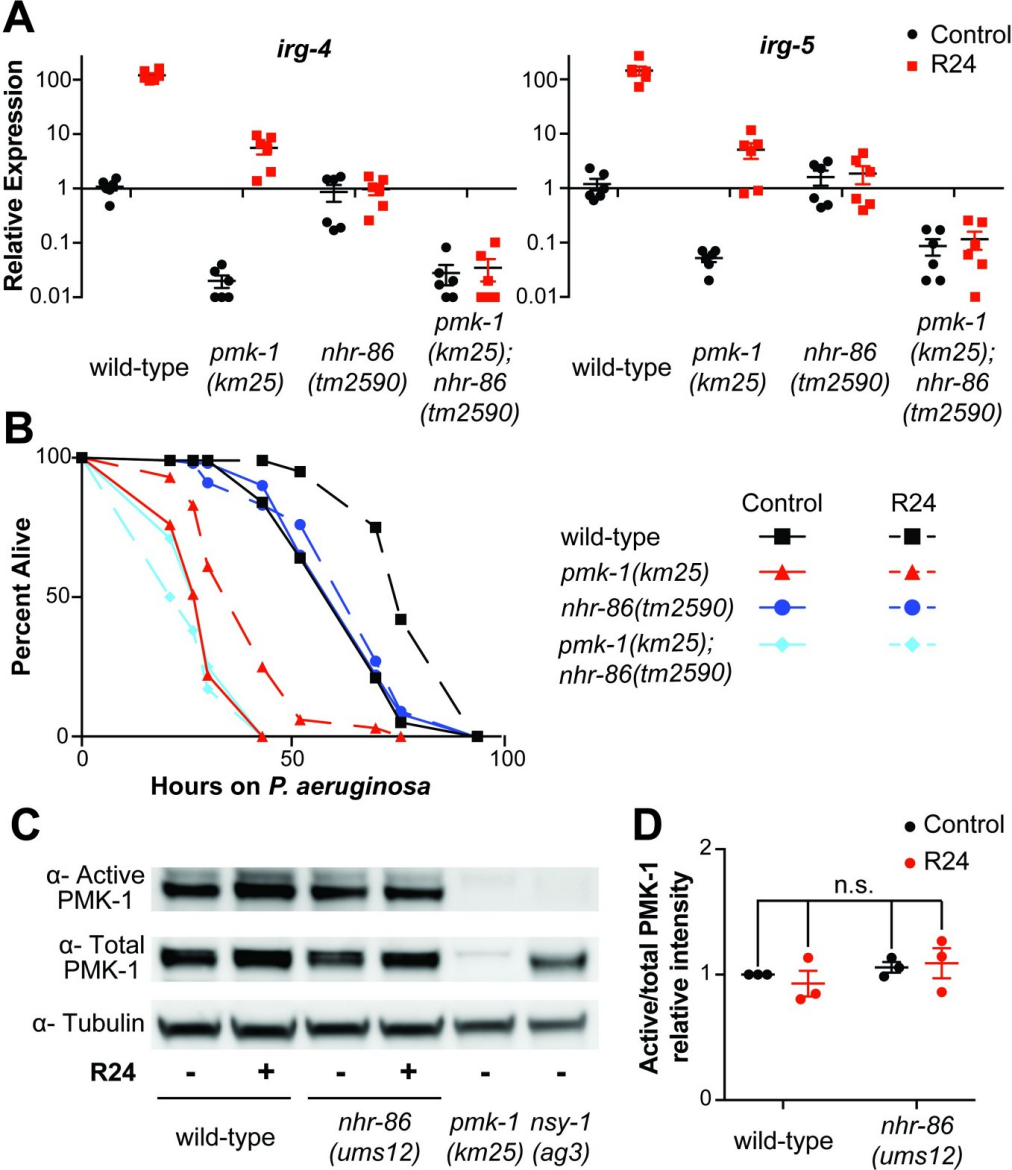
*nhr-86* induces innate immune defenses independent of the p38 MAPK *pmk-1*. **(A)** The expression of the *C*. *elegans* immune effector genes *irg-4*, *irg-5*, *drd-50*, *mul-1* and *irg-6* were analyzed by qRT-PCR in wild-type animals, *pmk-1(km25)*, *nhr-86(tm2590)*, and *pmk-1(km25); nhr-86(tm2590)* double mutants, each exposed to either R24 or the control for approximately 18 hours. Data for *drd-50*, *mul-1* and *irg-6* are shown in S5A Fig. Data are the average of six independent replicates, each normalized to a control gene with error bars representing SEM. Data are presented as the value relative to the average expression from all replicates of the indicated gene in the baseline condition (wild-type animals exposed to control). The difference in induction of *irg-4*, *irg-5*, *drd-50*, *mul-1* and *irg-6* by R24 in wild-type animals compared to each of the mutant strains is significant (p<0.0001 by 2-way ANOVA with Bonferroni multiple comparisons test). There is no significant difference between the expression of these genes in *pmk-1(km25)* animals exposed to control compared to either condition in the *pmk-1(km25); nhr-86(tm2590)*. **(B)** *P*. *aeruginosa* infection assays of *C*. *elegans* wild-type, *pmk-1(km25)*, *nhr-86(tm2590)*, and *pmk-1(km25); nhr-86(tm2590)*, each exposed to control or R24, are shown. The difference in susceptibility to *P*. *aeruginosa* between control and R24-exposed wild-type and *pmk-1(km25)* animals is significant (p<0.001). There is no significant difference between control and R24-exposed *nhr-86(tm2590)* and *pmk-1(km25); nhr-86(tm2590)* animals. Data are representative of three trials. Sample sizes, mean lifespan, % lifespan extension conferred by R24 treatment in each background and p values for all trials are shown in S4B Table. Significance was determined using Kaplan-Meier survival curves and log-rank tests. **(C)** Immunoblot analysis of lysates from L4 stage animals of the indicated genotype using antibodies that recognize the doubly-phosphorylated TGY motif of PMK-1 (⍺-Active PMK-1), the total PMK-1 protein (⍺-Total PMK-1) and tubulin (⍺-Tubulin). The total PMK-1 antibody detects total, but not active (phosphorylated) PMK-1. **(D)** The relative intensity of active PMK-1 and total PMK-1 was quantified from three biological replicates and is expressed as the average ratio of active to total PMK-1, relative to wild-type control. Error bars report SEM. There is no significant difference (n.s.) between these conditions (2-way ANOVA with Bonferroni multiple comparisons test).

The immunostimulatory molecule R24 upregulates innate immune effectors whose basal expression requires the p38 MAPK *pmk-1* [16], a key signaling mediator in a pathway that is critically important for host defense against bacterial pathogens [8, 19]. To determine if *nhr-86* and *pmk-1* function in the same or distinct pathways in the transcriptional modulation of innate immune effector genes, we compared gene expression (Figs 5A and S5A) and pathogen resistance (Fig 5B) phenotypes of the *pmk-1(km25)* and *nhr-86(tm2590)* single mutants with the *pmk-1(km25); nhr-86(tm2590)* double mutant. We previously observed that R24 can extend the lifespan of *pmk-1(km25)* mutant animals that are infected with *P*. *aeruginosa* [[16] and Fig 5B]. In addition, we found that *pmk-1* is dispensable for the induction of a group of innate immune effectors, including *irg-4*, *irg-5*, *mul-1* and *drd-50* [[16], see also Figs 5A and S5A]. However, because the basal level of expression of these four effectors is decreased in the *pmk-1(km25)* mutant, the absolute level of immune effector expression following exposure to R24 is markedly lower compared to controls (Figs 5A and S5A). The deficiency in the basal regulation of immune effectors in the *pmk-1(km25)* mutant contributes to the enhanced susceptibility of this mutant to *P*. *aeruginosa* infection in both naive and R24-treated animals [8, 19] (Fig 5B). These data indicate that R24 drives the induction of a protective immune response independent of *pmk-1*. Consistent with this observation, exposure to R24 does not cause an increase in the percentage of active (phosphorylated) PMK-1 relative to total PMK-1 in wild-type or *nhr-86(ums12)* animals (Fig 5C and 5D).

The susceptibility of the *pmk-1(km25); nhr-86(tm2590)* double mutant to *P*. *aeruginosa* infection in the absence of R24 is identical to the *pmk-1(km25)* mutant, further suggesting that NHR-86 functions in an R24-dependent manner (Fig 5B). Importantly, the *nhr-86(tm2590)* allele suppressed the R24-mediated enhanced longevity in the *pmk-1(km25)* background (Fig 5B). Accordingly, the basal expression of *irg-4*, *irg-5*, *mul-1*, *drd-50* and *irg-6* is reduced in the *pmk-1(km25); nhr-86(tm2590)* double mutant to the same level as the *pmk-1(km25)* mutant (Figs 5A and S5A). Importantly, the R24-mediated induction of these immune effectors in the *pmk-1(km25)* background is blocked by the *nhr-86(tm2590)* mutation (Figs 5A and S5A).

Of note, the induction of at least two cytochrome P450 xenobiotic detoxification genes by R24 is not dependent on either *nhr-86* or *pmk-1* (S5B Fig). These data further support that *nhr-86* is required for only a specific subset of the R24-induced genes (Fig 2).

**Fig 6:**
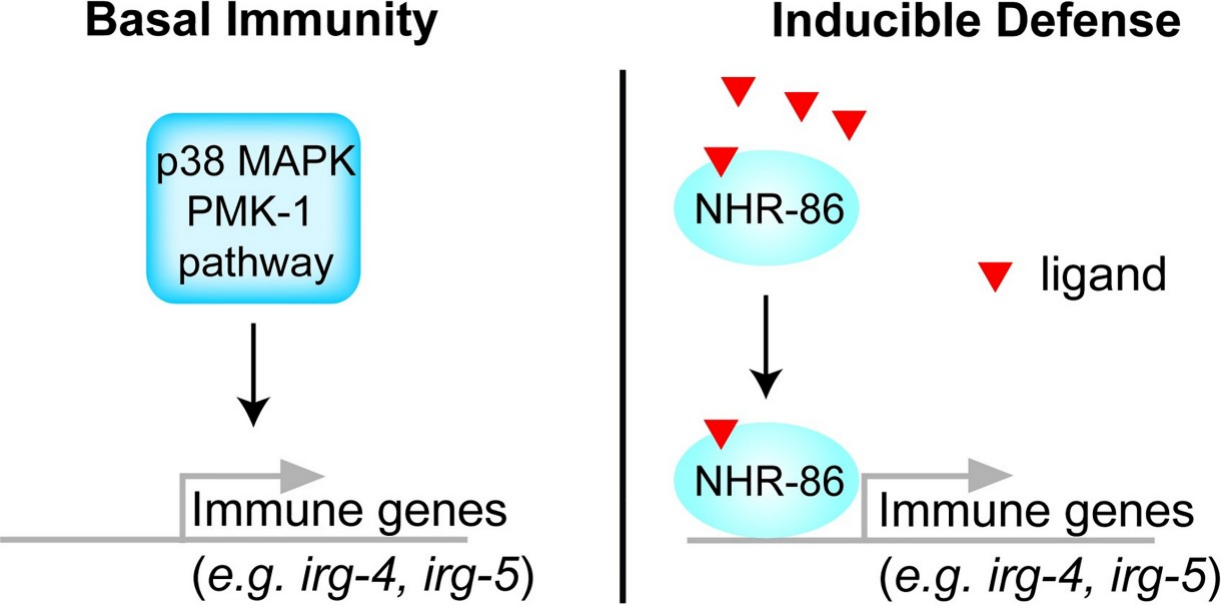
Model of NHR-86-mediated immune regulation in *C*. *elegans*. The basal expression of immune effectors such as *irg-4*, *irg-5*, *mul-1* and *drd-50* are ensured by p38 MAPK PMK-1. Activated NHR-86 traffics to the promoters of these and other immune effectors to drive their induction and provide protection from bacterial infection.

In summary, these genetic epistasis experiments support the model that, upon activation, NHR-86 traffics to the promoters of immune effectors to mount a protective immune response in a manner independent of the p38 MAPK *pmk-1* pathway (Fig 6). In this context, a principal role of the p38 MAPK *pmk-1* is to ensure basal resistance to pathogens by controlling the tonic expression of innate immune effectors, such as *irg-4*, *irg-5*, *mul-1* and *drd-50*.

## Discussion

This study extends the known functions of *C*. *elegans* NHRs to include the activation of anti-pathogen transcriptional responses. Following treatment with an immunostimulatory small molecule, NHR-86 directly activates innate immune effector transcription in a manner that promotes resistance to bacterial infection. ChIP-seq and mRNA-seq revealed an enrichment for innate immune effectors among the transcriptional targets of NHR-86, including at least three genes, *irg-4*, *irg-5* and *irg-6*, that are each required for normal resistance to *P*. *aeruginosa* infection. Consistent with this model, the induction of protective immune defenses by NHR-86 occurs independently of the p38 MAPK *pmk-1*. In addition, in the absence of an immunostimulatory molecule, NHR-86 is not required for the basal regulation and is not at the promoters of immune effectors. Arda *et al*. proposed that NHRs, and NHR-86 in particular, organize modular gene regulatory networks to facilitate the rapid coordination of adaptive responses to intracellular ligands [3]. Our data show that an anti-pathogen transcriptional response is one such adaptive response.

We previously demonstrated that a conserved component of the Mediator transcriptional regulatory complex, MDT-15/MED15, links detoxification and innate immune defenses in *C*. *elegans* [17]. The Mediator complex is conserved from yeasts to humans and regulates transcription by physically interacting with both transcriptional regulators and RNA polymerase II [30, 31]. Individual mediator subunits, particularly those like MDT-15, which are in the tail region of the complex, dictate the physical interactions with transcriptional regulators and play important roles in modulating specific transcriptional outputs [30–34]. Like *nhr-86*, *mdt-15* is required for the induction of immune effectors whose basal expression is dependent on the p38 MAPK PMK-1 pathway [17]. In addition, MDT-15 functions downstream of the PMK-1 cascade to control the expression of immune effectors [17]. Notably, a subset of the immune effectors in *mdt-15*-deficient animals, including *irg-4*, *irg-5*, and *drd-50* have reduced basal levels of expression [as in *pmk-1(km25)* mutants] and cannot be induced by the small molecule R24 (as in *nhr-86* loss-of-function mutants). Importantly, NHR-86 is known to physically interact with MDT-15 [3]. Thus, we hypothesize that MDT-15 and NHR-86 function together to drive the transcription of immune response genes, such as *irg-4*, *irg-5* and *drd-50*.

The ligand that activates NHR-86 is not known. Indeed, it is possible that R24 or a metabolite derived from this compound is an activating ligand of NHR-86. However, it is important to note that not all R24-induced genes are dependent on *nhr-86* for their upregulation. Alternatively, NHR-86 may detect a host-derived ligand that is associated with the toxic effects of R24 on nematode cells. R24 induces xenobiotic detoxification genes and shortens the lifespan of nematodes growing in standard laboratory conditions [16]. *C*. *elegans* activates immune defenses following toxin-mediated disruption of cellular homeostasis [35]. Thus, NHR-86 may function as part of a similar cellular surveillance mechanism, although this is not known. Notably, *nhr-86* loss-of-function mutants are not more susceptible to *P*. *aeruginosa* infection at baseline. While *nhr-86* is required for the induction of the immune effectors *irg-5* and *irg-6* by the immunostimulatory xenobiotic R24, it is dispensable for their induction during *P*. *aeruginosa* infection. Thus, it is possible that *P*. *aeruginosa* infection does not produce a ligand that is sensed by NHR-86 or there are redundant mechanisms engaged to activate *C*. *elegans* defenses during pseudomonal infection. In either case, our data demonstrate that a *C*. *elegans* NHR can drive a protective transcriptional response towards a bacterial pathogen. These findings raise the possibility that NHRs provide a facile and evolutionarily adaptable mechanism to activate protective immune defenses in response to diverse ligands.

## Materials and methods

### *C*. *elegans* and bacterial strains

*C*. *elegans* strains were maintained on standard nematode growth media plates with *E*. *coli* OP50 as a food source, as described [36]. The previously published *C*. *elegans* strains used in this study were: N2 Bristol [36], KU25 *pmk-1(km25)* [8], AU306 *agIs44* [P*irg-4*::*GFP*::*unc-54-3’UTR; Pmyo-2*::*mCherry*] [17], AY101 *acIs101* [p*DB09*.*1(Pirg-5*::*gfp)*; p*RF4(rol-6(su1006))*] [21], SJ405 *zcIs4 (Phsp-4*::*gfp)* [37], VL491 *nhr-86(tm2590)* [3], and VL648 *unc-119(ed3)*; *wwIs22 [Pnhr-86*::*nhr-86ORF*::*GFP unc-119(+)]* [3]. The strains developed in this study were: RPW137 *nhr-86(ums12)*, RPW119 *pmk-1(km25);nhr-86(tm2590)*, RPW99 *nhr-86(tm2590); agIs44*, RPW106 *nhr-86(tm2590)*; *acIs101*, and RPW165 *nhr-86(ums12); agIs44*. *Pseudomonas aeruginosa* strain PA14 was used for all studies [38].

### *C*. *elegans* strain construction

CRISPR/Cas9 was used to generate *nhr-86(ums12)* as described [39]. Target sequences were selected on exons 1 and 6 of *nhr-86*. Forward and reverse oligonucleotides were designed to contain the target sequence and overhangs compatible with *Bsa*I sites in plasmid pPP13, a modified version of pRB1017 [39, 40]. Forward and reverse oligonucleotides were annealed and ligated into pPP13 cut with *Bsa*I to create the gRNA plasmids. Plasmids were confirmed by sequencing. A DNA mixture of pDD162 (50 ng/L), the gRNA plasmids (25 ng/L each), pJA58 (50 ng/L) and the ssODN repair template for *dpy-10(cn64)* (20 ng/L) was prepared in injection buffer (20 mM potassium phosphate, 3 mM potassium citrate, 2% PEG, pH 7.5) and injected into N2 worms. Mutations in the *dpy-10* gene were used as a CRISPR co-conversion marker. The F1 progeny were screened for Rol and Dpy phenotypes 3–4 days after injection and then for deletions in the *nhr-86* coding region using PCR. The *nhr-86(ums12)* mutant contains a 5539 bp deletion that spans from 17 bp upstream of the ATG to 30 bp before the stop codon with an insertion of 6 bp at the breakpoint. Primer sequences used for genotyping are listed in S5 Table.

### Feeding RNAi screen

A previously-described library containing RNAi clones corresponding to 258 of the 284 NHRs in the *C*. *elegans* genome was used for this study [14]. These genes were screened for their ability to abrogate the induction of *agIs44* by 70 μM R24, as described [17].

### *C*. *elegans* bacterial infection and other assays

“Slow killing” *P*. *aeruginosa* infection experiments were performed as previously described [18, 41]. In all of these assays, the final concentration of DMSO was 1% and 70 μM R24 was used. Wild-type is either N2 or *agIs44*. All pathogenesis and lifespan assays are representative of three biological replicates. Sample sizes, mean lifespan, % lifespan extension conferred by R24 treatment in each background (where applicable) and p values for all trials are shown in S4 Table.

### mRNA-seq, NanoString ncounter gene expression analyses and qRT-PCR

Synchronized, L1 stage, hermaphrodites *C*. *elegans* of the indicated genotypes were grown to the L4/ young adult stage, transferred to assay plates, and incubated at 20°C overnight. 70 μM R24 or solvent control (DMSO, 1% final concentration) assay plates were prepared as described [16–18]. RNA was isolated using TriReagent (Sigma-Aldrich), purified on a column (Qiagen), and analyzed by mRNA-seq using the BGISEQ-500 platform (BGI Americas Corp). mRNA-seq data analysis was performed by BGI Americas Corp. Biological replicate RNA samples were analyzed using NanoString nCounter Gene Expression Analysis (NanoString Technologies) with a “codeset” designed by NanoString that contained probes for 118 *C*. *elegans* genes. The codeset has been described previously [17, 18]. Probe hybridization, data acquisition and analysis were performed according to instructions from NanoString with each RNA sample normalized to the control genes *snb-1*, *ama-1* and *act-1*. For the qRT-PCR studies, RNA was reverse transcribed to cDNA using the RETROscript Kit (Life Technologies) and analyzed using a CFX1000 machine (Bio-Rad). The sequences of primers that were designed for this study are presented in S5 Table. Other primers were previously published [19, 27, 33, 42]. All values were normalized against the control gene *snb-1*. Fold change was calculated using the Pfaffl method [43].

### Immunoblot analyses

*C*. *elegans* were prepared as described above to ensure that stage-matched, hermaphrodite animals at the young L4 larval stage were studied in each condition. Protein lysates were prepared as previously described [17] and probed with a 1:1000 dilution of an antibody that recognizes the doubly-phosphorylated TGY motif of PMK-1 (Promega Corporation). Monoclonal anti-α-tubulin antibody was used at a dilution of 1:1,000 (Sigma-Aldrich). A polyclonal antibody against the total PMK-1 protein was raised using the peptide DFQKNVAFADEEEDEEKMES (PMK-1 amino acids 358 to 377) in a rabbit (Thermo Scientific Pierce Custom Antibody Services) and used at a dilution of 1:1000. We confirmed that the total PMK-1 antibody detects total, but not active (phosphorylated) PMK-1 (Fig 5C). Horseradish peroxidase (HRP)-conjugated anti-rabbit (Cell Signaling Technology) and anti-mouse IgG secondary antibodies (Abcam) were diluted 1:10,000 and used to detect the primary antibodies following the addition of ECL reagents (Thermo Fisher Scientific, Inc.), which were visualized using a BioRad ChemiDoc MP Imaging System. The band intensities were quantified using BioRad Image Lab software version 5.2.1, and the ratio of active phosphorylated PMK-1 to total PMK-1 was calculated with all samples normalized to the ratio of wild-type control animals.

### ChIP-qPCR, ChIP-seq and bioinformatics

Chromatin immunoprecipitation was performed with a strain containing a GFP-tagged NHR-86 protein (NHR-86::GFP) that has been previously characterized [3]. *nhr-86* transcript levels are 2.7-fold elevated in the NHR-86::GFP strain compared to wild-type (S2B Fig). ChIP was performed as previously described [44, 45] with modifications. Briefly, L4 synchronized, hermaphrodite *C*. *elegans* (wild-type and transgenic NHR-86::GFP animals) were exposed to “slow killing” plates [41] containing either DMSO (1%) or 70 μM R24 for approximately 18 hours. Animals were then collected and washed with 4°C M9 and phosphate-buffered saline to remove bacteria. Cross-linking of protein and DNA was performed in 2% formaldehyde for 30 minutes at room temperature. Cross-linking was quenched with 100 mM glycine, animals were washed in M9, resuspended in lysis buffer (50 mM Hepes–KOH pH 7.5, 300 mM NaCl, 1 mM EDTA, 1% (v/v) Triton X-100, 0.1% (w/v) sodium deoxycholate, 0.5% (v/v) N-Lauroylsarcosine, and protease inhibitors) and lysed with a Teflon homogenizer. Lysates were then sonicated using a Bioruptor UCD-200 for 10 cycles (30 s on, 30 s off) to obtain 500–1000 bp DNA fragments. Sonicated lysates (2 mg) were pre-cleared with protein G Dynabeads (Invitrogen), 10% of lysate removed for input, and incubated with 5 μg anti-GFP antibody (Roche) overnight. Immune complexes were collected with protein G Dynabeads, washed, and eluted from beads. Cross-links were reversed at 65°C overnight and DNA fragments were purified with PCR purification columns (Qiagen). qPCR was performed on input and immunoprecipitated samples using primers designed around the transcription start site. All ChIP data are presented as percent input normalized to a random intragenic region on chromosome four. Primer sequences are available in S5 Table.

ChIP-seq was performed by BGI Americas Corp. The raw sequencing data were first clipped for adaptor sequences and then mapped to the *C*. *elegans* genome (ce10, UC Santa Cruz) by the Burrows-Wheeler Aligner algorithm (BWA MEM, BWA version 0.7.15). The output SAM files were processed and sorted with the Picard tools. The output mapping files (BAM files) were filtered with SAMtools to remove any read that had a mapping quality less than 10 (SAMtools view–b–q 10 input.bam > output.bam). Peaks were determined using MACS version 2.1 with the no-model parameter. The final set of peaks were called if the difference in intensity values of samples had a significance level of p-value < 0.025.

To identify candidate motifs for NHR-86 binding, ChIP peaks that were located in promoter regions of genes were examined using the MEME motif analysis platform [Parameters: minw = 8, maxw = 25, in two modes (zoops & anr), significance threshold (E-value > = 1e-01), http://meme.sdsc.edu]. A background model is used by MEME to calculate the log likelihood ratio and statistical significance of the motif. We set the following requirements: the most significant motif should exist in 50% of input sequences, and the genes containing the motif should have the largest overlap between ChIP-Seq and RNA-seq datasets. A single 15 bp motif was identified that met these criteria (E-value: 1.7e-003, S2C Fig). 66 sites of 101 input sequences had this motif, including 15 of the 32 genes that overlapped in the ChIP-Seq and RNA-seq datasets.

### Microscopy

Nematodes were mounted onto agar pads, paralyzed with 10 mM levamisole (Sigma) and photographed using a Zeiss AXIO Imager Z2 microscope with a Zeiss Axiocam 506mono camera and Zen 2.3 (Zeiss) software.

### Statistical analyses

Differences in survival of *C*. *elegans* in the *P*. *aeruginosa* pathogenesis assays were determined with the log-rank test using OASIS 2 as previously described [46]. Data from one experiment that is representative of the replicates is shown. Other statistical tests, indicated in the figure legends, were performed using Prism 7 (GraphPad Software).

## Supporting information

S1 Fig An RNAi screen identifies a role for the nuclear hormone receptor *nhr-86* in the induction of *C*. *elegans* immune effectors. **(A)** qRT-PCR data of *nhr-86* mRNA in the *nhr-86(ums12)* mutant. **(B)** qRT-PCR data of *irg-6* as described in Fig 1. In A and B, data are the average of three or four independent replicates, respectively, each normalized to a control gene with error bars representing SEM. Data are presented as the value relative to the average expression from all replicates of the indicated gene in the baseline condition (wild-type animals exposed to control). **(C)** *P*. *aeruginosa* pathogenesis assay and **(D)** lifespan on *E*. *coli* OP50 of animals exposed to the indicated RNAi bacteria. Data are representative of three trials. Sample sizes, mean lifespan and p values for all trials are shown in S4C and S4D Table. Significance was determined using Kaplan-Meier survival curves and log-rank tests.
(TIF)

S2 Fig NHR-86 binds to the promoters of innate immune genes to drive their transcription. qRT-PCR was used to measure **(A)** *irg-4* and **(B)** *nhr-86* in animals of the indicated genotypes. Data are the average of three independent replicates, each normalized to a control gene with error bars representing SEM. Data are presented as the value relative to the average expression from all replicates of the indicated gene in the baseline condition (wild-type animals exposed to control in A and wild-type in B). **(C)** The 15-bp sequence that was enriched in the promoters that were bound by NHR-86::GFP.
(TIF)

S3 Fig The immune response induced by *nhr-86* protects *C*. *elegans* from *P*. *aeruginosa* infection. **(A)** *Phsp-4*::*GFP*, **(B)** *Pirg-4*::*GFP* and **(C)** *Phsp-4*::*GFP* animals were exposed to the indicated RNAi conditions and treated with DMSO (control) or 10 μg/mL tunicamycin (TC) overnight at 20°C and photographed. Red expression in *Pirg-4*::*GFP* animals is the *Pmyo-2*::*mCherry* co-injection marker. Scale bar equals 100 μm.
(TIF)

S4 Fig *nhr-86(ums12)* does not abrogate the induction of *irg-1*, *irg-5* or *irg-6* during *P*. *aeruginosa* infection. qRT-PCR data of *irg-1*, *irg-4*, *irg-5* and *irg-6* in wild-type or *nhr-86(ums12)* animals exposed to *E*. *coli* or *P*. *aeruginosa* for 6 hours. * equals p<0.05 for the difference in expression of the indicated gene between wild-type and *nhr-86(ums12)* in the *P*. *aeruginosa*-exposed condition. All other differences were not significant. Data are presented relative to uninfected wild-type animals.
(TIF)

S5 Fig *nhr-86* induces innate immune defenses independent of the p38 MAPK *pmk-1*. qRT-PCR data of *drd-50*, *mul-1 and irg-6* **(A),** and *cyp-35B2* and *cyp-35A3* **(B)** as described in Fig 5A.
(TIF)

S1 Table Genes identified in an RNAi screen for NHRs that control the induction of *Pirg-4*::*GFP* by R24. (XLSX)

S2 Table *nhr-86-*dependent genes from the mRNA-seq experiment. (XLSX)

S3 Table ChIP-seq data showing the promoters that were bound by NHR-86. (XLSX)

S4 Table Sample sizes, mean lifespan, % lifespan extension conferred by R24 treatment in each background and p values for the *C*. *elegans* pathogenesis and lifespan assays. **(A)** Data for Fig 4A. **(B)** Data for Fig 5B. **(C)** Data for S1C Fig. **(D)** Data for S1D Fig.
(XLSX)

S5 Table Primer sequences that were designed for this study. (XLSX)
