# Supplementary figures and images for: The nuclear hormone receptor NHR-86 controls anti-pathogen responses in C. elegans
Source: PLoS Genet. 2019 Jan 22;15(1):e1007935. doi: 10.1371/journal.pgen.1007935 (PMC6358101; doi:10.1371/journal.pgen.1007935)

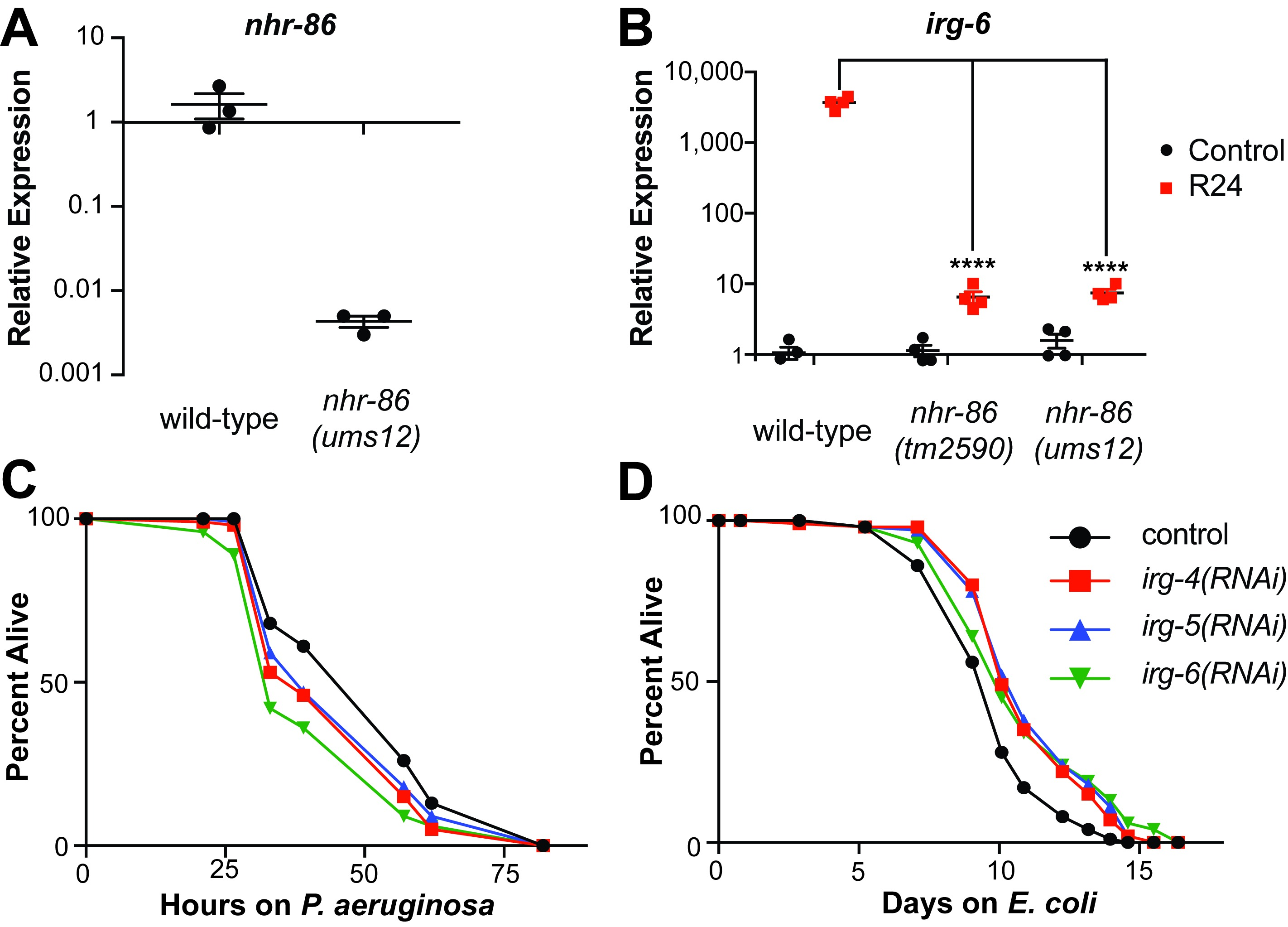

Supplement: S1 Fig — (A) qRT-PCR data of nhr-86 mRNA in the nhr-86(ums12) mutant. (B) qRT-PCR data of irg-6 as described in Fig 1. In A and B, data are the average of three or four independent replicates, respectively, each normalized to a control gene with error bars representing SEM. Data are presented as the value relative to the average expression from all replicates of the indicated gene in the baseline condition (wild-type animals exposed to control). (C) P. aeruginosa pathogenesis assay and (D) lifespan on E. coli OP50 of animals exposed to the indicated RNAi bacteria. Data are representative of three trials. Sample sizes, mean lifespan and p values for all trials are shown in S4C and S4D Table. Significance was determined using Kaplan-Meier survival curves and log-rank tests. (TIF) [file pgen.1007935.s001.tif]

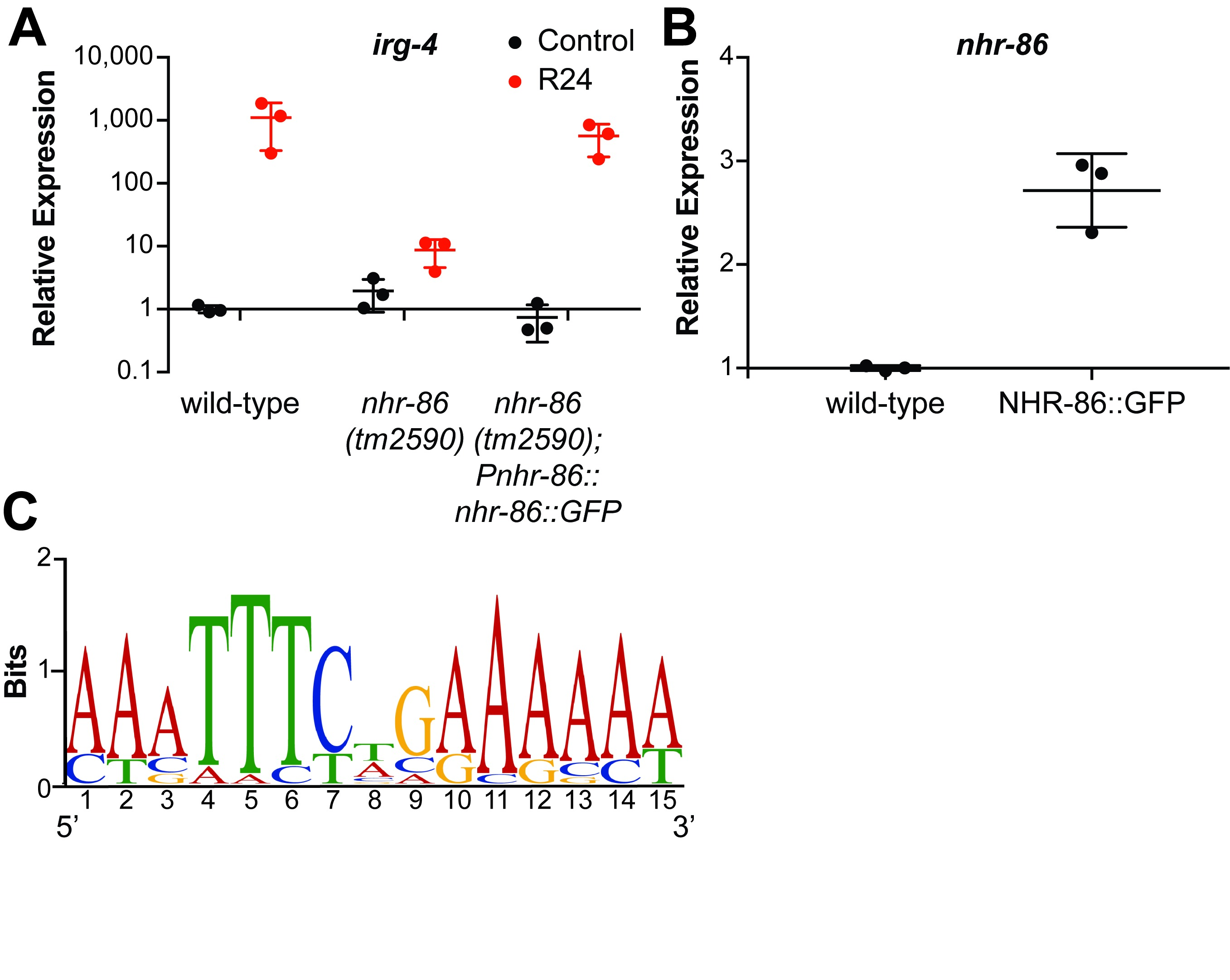

Supplement: S2 Fig — qRT-PCR was used to measure (A) irg-4 and (B) nhr-86 in animals of the indicated genotypes. Data are the average of three independent replicates, each normalized to a control gene with error bars representing SEM. Data are presented as the value relative to the average expression from all replicates of the indicated gene in the baseline condition (wild-type animals exposed to control in A and wild-type in B). (C) The 15-bp sequence that was enriched in the promoters that were bound by NHR-86::GFP. (TIF) [file pgen.1007935.s002.tif]

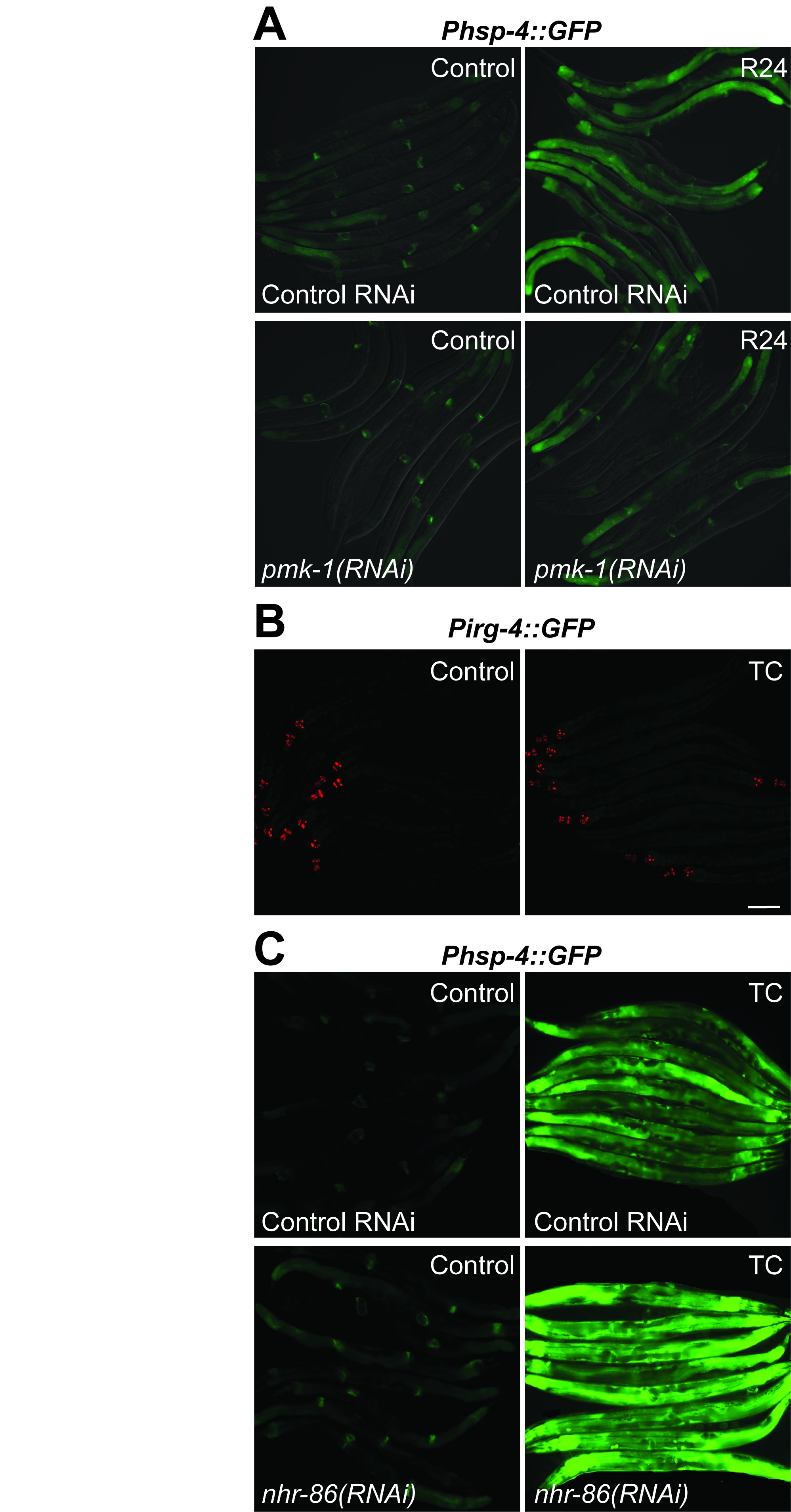

Supplement: S3 Fig — (A) Phsp-4::GFP, (B) Pirg-4::GFP and (C) Phsp-4::GFP animals were exposed to the indicated RNAi conditions and treated with DMSO (control) or 10 μg/mL tunicamycin (TC) overnight at 20°C and photographed. Red expression in Pirg-4::GFP animals is the Pmyo-2::mCherry co-injection marker. Scale bar equals 100 μm. (TIF) [file pgen.1007935.s003.tif]

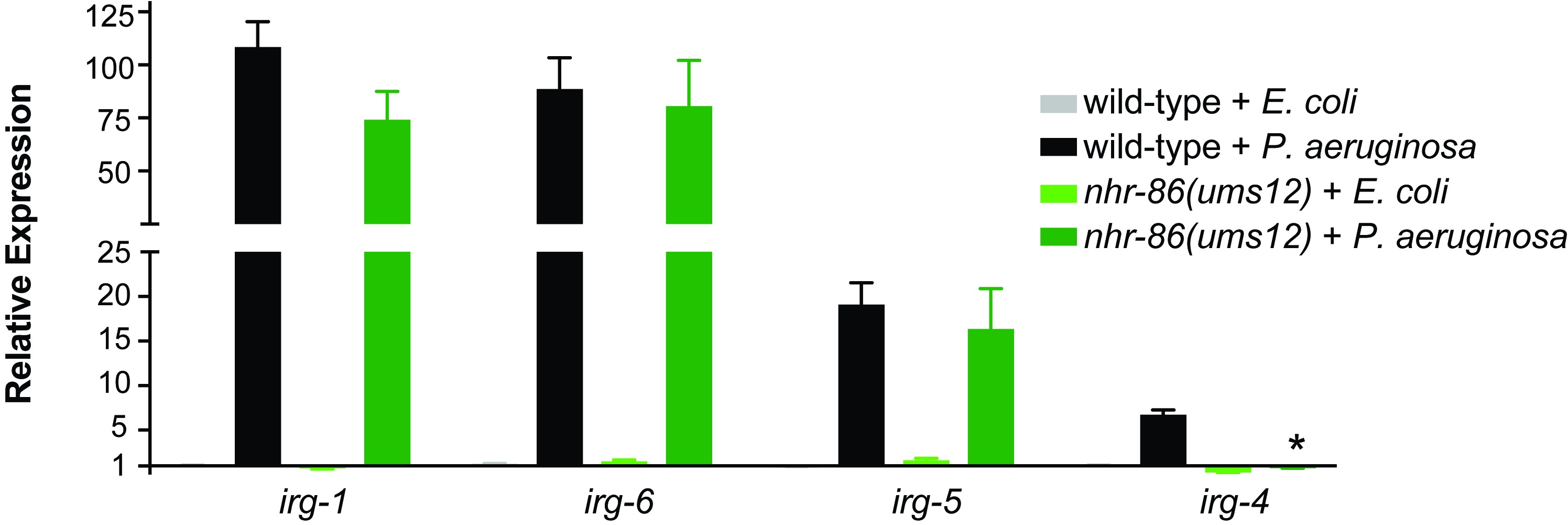

Supplement: S4 Fig — qRT-PCR data of irg-1, irg-4, irg-5 and irg-6 in wild-type or nhr-86(ums12) animals exposed to E. coli or P. aeruginosa for 6 hours. * equals p<0.05 for the difference in expression of the indicated gene between wild-type and nhr-86(ums12) in the P. aeruginosa-exposed condition. All other differences were not significant. Data are presented relative to uninfected wild-type animals. (TIF) [file pgen.1007935.s004.tif]

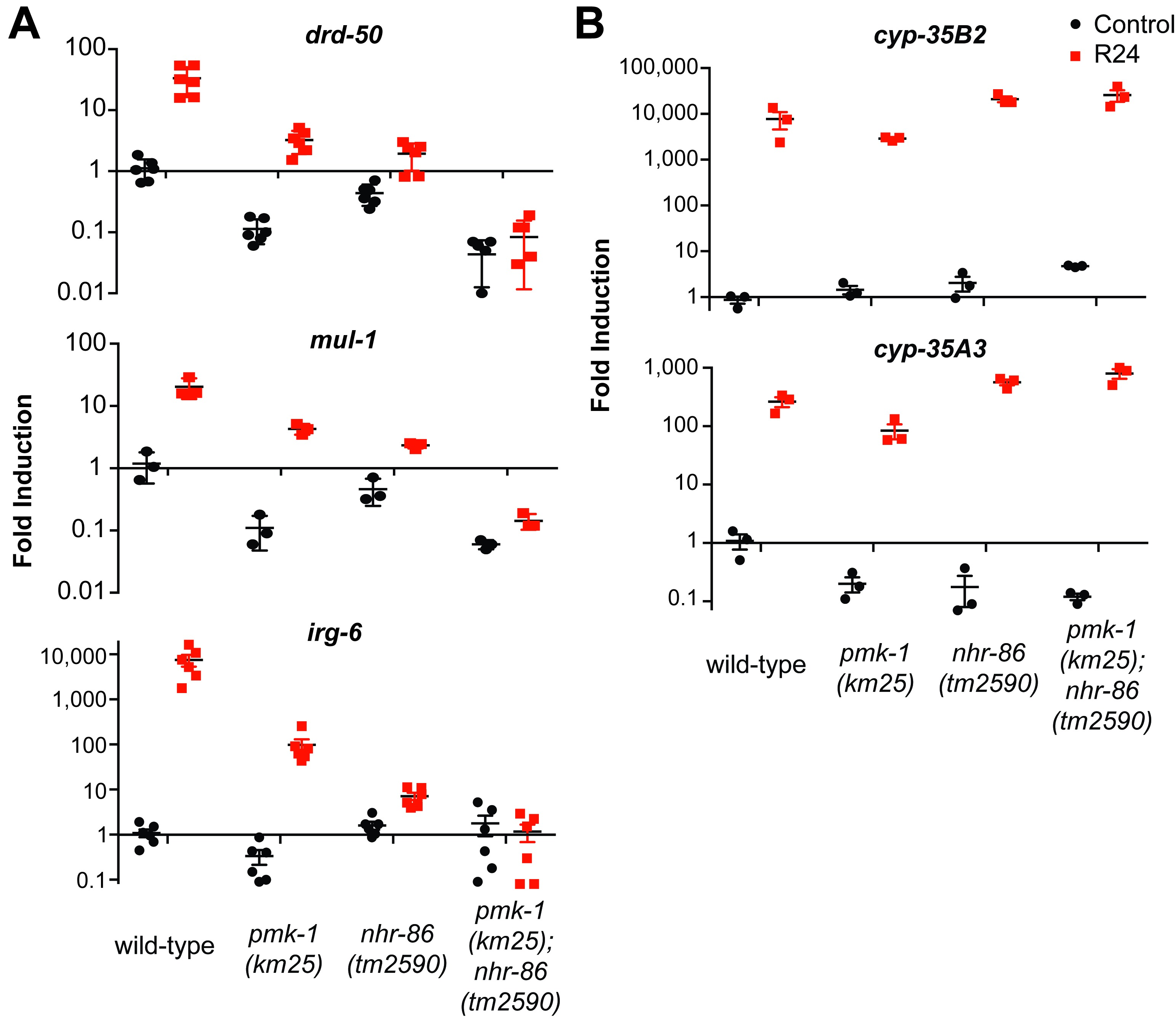

Supplement: S5 Fig — qRT-PCR data of drd-50, mul-1 and irg-6 (A), and cyp-35B2 and cyp-35A3 (B) as described in Fig 5A. (TIF) [file pgen.1007935.s005.tif]
